# Long Term Results of Two-Stage Revision for Chronic Periprosthetic Hip Infection: A Multicenter Study

**DOI:** 10.3390/jcm11061657

**Published:** 2022-03-16

**Authors:** Beau J. Kildow, Bryan D. Springer, Timothy S. Brown, Elizabeth Lyden, Thomas K. Fehring, Kevin L. Garvin

**Affiliations:** 1 Department of Orthopaedic Surgery and Rehabilitation, University of Nebraska Medical Center, Omaha, NE 68198, USA; elyden@unmc.edu (E.L.); kgarvin@unmc.edu (K.L.G.); 2 Department of Orthopaedic Surgery, OrthoCarolina, Charlotte, NC 27707, USA; bryan.springer@orthocarolina.com (B.D.S.); thomas.fehring@orthocarolina.com (T.K.F.); 3 Department of Orthopaedic Surgery, Houston Methodist Orthopedics & Sports Medicine, 6445 Main Street Suite 2500, Houston, TX 77030, USA; timothy.s.brown@ui.edu

**Keywords:** two-stage exchange, periprosthetic joint infection, long-term, arthroplasty

## Abstract

Background: Two-stage exchange arthroplasty remains the gold standard in the United States for treatment of chronic periprosthetic joint infection (PJI). Long-term reinfection rates and clinical outcomes with sufficient subject numbers remain limited. The purpose was to evaluate the long-term outcomes following two-stage exchange following hip arthroplasty. Methods: Retrospective review of 221 patients who underwent two-stage exchange hip arthroplasty for chronic PJI at three large tertiary referral institutions from 1990–2015. Outcomes including reinfection, mortality, and all-cause revision were calculated. Cumulative incidence of reinfection with death as competing factor was also calculated. Risk factors for reinfection were determined using Cox multivariate regression analysis. Results: Rate of infection eradication and all-cause revision was 88.24% and 22.6%, respectively. Overall mortality rate was 40.72%. Patients with minimum five-year follow-up (*n* = 129) had a success rate of 91.47% with mortality rate of 41.1%. Major risk factors for reinfection included polymicrobial infection (HR = 2.36, 95% CI: 1.08–5.14) and antibiotic resistant organism (HR = 2.36, 95% CI: 1.10–5.04). Conclusion: This is the largest series with greater than 5-year follow-up evaluating outcomes of two-stage exchange hip arthroplasty. This technique resulted in a relatively high infection eradication, however, the mortality rate is alarmingly high. Antibiotic resistant organisms appear to be highest risk factor for failure.

## 1. Introduction

Periprosthetic joint infection (PJI) remains as one of the most common major complications following joint replacement surgery [1]. PJI’s will increase exponentially to 10,000 cases per year by 2030 [2]. With the almost exponential increase in joint replacement surgery, PJI will be costly and straining for the surgeon and healthcare system. 

Two-stage exchange arthroplasty is the gold standard for the treatment of chronic PJI [3,4]. This process involves the placement of a temporary antibiotic spacer with a period of intravenous antibiotics followed by reconstruction when infection is determined to be eradicated. The reported success rates in the literature have varied between 65% and 95% depending on the definition of success as well as other factors. For example, many studies have chosen to exclude patients who died prior to 2-years of follow-up [5,6], when high mortality rates have been reported following this treatment protocol [7,8]. Factors such as host and extremity grade and organism virulence also affect the outcome of reinfection [9,10,11,12]. There are few studies [7,13,14] reporting two-stage hip exchange outcomes. At many institutions, two-stage exchange remains the standard of care for the management of chronic PJI. 

The purpose is to evaluate long-term survival and infection eradication after two-stage hip exchange arthroplasty at large tertiary referral centers. We also investigate potential risk factors related with reinfection.

## 2. Methods

**Figure 1:**
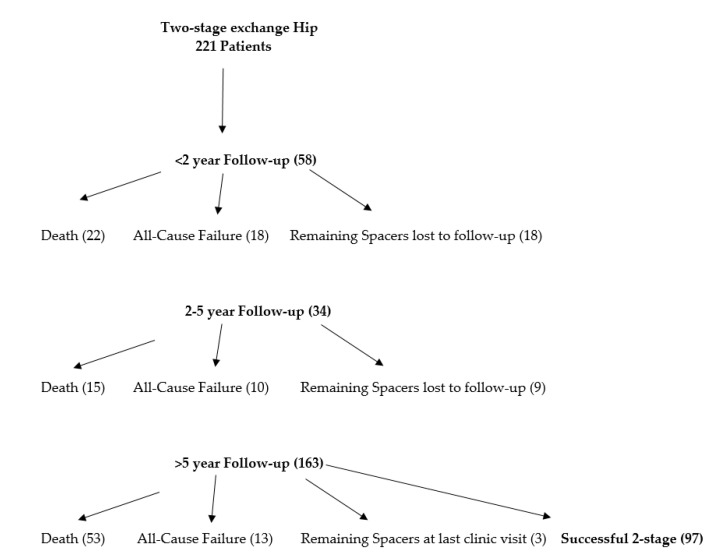
Patient flowchart.

Institutional databases from three large tertiary referral hospitals were used to collect a list of patients who underwent the first stage in a planned two-stage hip exchange arthroplasty for chronic infection from 1990–2015. Patients who presented with an antibiotic spacer from an outside institution were excluded. During this retrospective review timeframe, two-stage exchange was the treatment method across all institution for chronic PJI. Overall treatment protocol involved the insertion of a static or articulating hip spacer after thorough debridement. The period between stages involved the administration of intravenous (IV) antibiotics for six or more weeks. Surgeon discretion was used for timing of reimplantation. However, all institutions-based decisions on the decreasing value of erythrocyte sedimentation rate (ESR) and C-reactive protein (CRP). All patients who obtained the first-stage of planned two-stage procedure were included. Treatment protocols varied slightly between institutions. Infection diagnosis involved both clinical and laboratory acumen such as physical examination, past and present history, synovial fluid and inflammatory markers, and culture. After 2011, MSIS criteria was used to diagnosis PJI [15]. This study yielded 221 patients with an average follow-up of 6.14 years (death included). Patients who were considered lost to follow-up prior to two years or those who did not have a recent clinic visit were contacted via phone. No exclusions were made for lack of follow-up in an intention to treat model in order to construct a survival analysis, identify rate of attrition/mortality, as well as limit bias within the sample. Patients were then placed into categories for sub-analysis based on short-term (0–2 years), (2–5 years), and long-term (greater than five years) follow-up. (Figure 1. Flowchart). Medical reports were interrogated to obtain major medical conditions, age, and sex. Major comorbidities collected included coronary vascular disease, chronic lung and kidney disease, cirrhosis, history of myocardial infraction, congestive heart failure, cancer, systemic illness, immunocompromising illness, diabetes, and smoking prior to initiation of treatment. The pathogenic organism and potential resistance were recorded. Resistance was defined as one of the commonly identified “ESKAPE” microorganisms of resistance in PJI including: cephalosporin resistant *E. Faecium*, methicillin resistant *S. aureus*, multi-drug resistant *K. Pneumoniae*, *A. Baumannii*, and *P. aeruginosa*, cephalosporin resistant *Enterobacter* species, and methicillin resistant *S. epidermidis*. Surgical and medical complications were recorded during the study period. Mortality and the number of subjects who did not proceed to the two-stage procedure were calculated. Infection eradication was determined if patients did not have to return to for a surgical complication following reimplantation. Failure was defined as a complication besides infection that included implant failure such as dislocation or periprosthetic fracture that required return to the operating room that occurred during the first or second stage. Institutional review board approval was attained at all three institutions. 

### Statistical Analysis

Continuous and categorical data were calculated using standard descriptive statistics. Kaplan–Meier method was used to calculate survival analysis based on mortality and failure of reimplantation. Cumulative incidence methods was calculated using death as a competing event. Chi-squared or Fisher’s exact tests were used for categorical data. Independent t-tests or Mann–Whitney U tests were used for continuous data. Cox proportional hazards regression modeling reported with 95% confidence intervals were used to identify risk factors for reinfection. The Cox models were reported to account for the competing event of death. Significance was defined with a *p*-value of < 0.05. The software product SAS v. 9.4 (SAS Institute, Cary, NC, USA) was used for statistical analysis. 

## 3. Results

**Figure 2:**
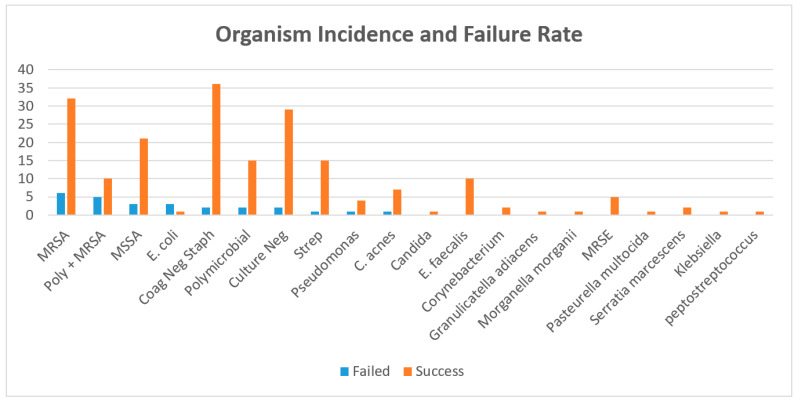
Organism Profile (%Failure). Associated MRSA infection yielded 20.75% failure rate. Abbreviations: Coag: coagulation, Neg: negative, MRSA: methicillin-resistant staphylococcus aureus, MSSA: methicillin-sensitive staphylococcus aureus, Strep: streptococcus species, Poly: polymicrobial, E. Faecalis: enterococcus faecalis, C. acnes: cutibacterium acnes, MRSE: methicillin-resistant staphylococcus epidermidis, and *E. coli*: *Escherichia coli*.

There were 221 patients included who underwent a planned two-stage exchange hip arthroplasty for chronic periprosthetic joint infection. There were 105 (47.51%) males and 116 (52.49%) females with an average age of 65.79 years. Infection eradication rate was 88.24% at mean 6.14 (range, 0.10–25.5) years follow-up. The overall complication rate was 19.45% (43/221), while 13.57% (30/221) were not reimplanted following the first stage. Patients that did not get reimplanted continued to retain spacer construct on final follow-up without any complication or failure. Reasons included: medically not safe for reimplantation (8), lost to follow-up after last clinic visit (5), and elected not to be reimplanted (17). There were seven complications for implant failure after reimplantation requiring surgical revision: dislocation (5) and periprosthetic fracture (2). Overall mortality rate was 40.72% (90/221) (Table 1). Ranges of eradication and mortality rates across all institutions was (87.40–89.80%) and (38.10–42.10%), respectively. The most common microbe was coagulase-negative staphylococcus, however, was successfully treated with an eradication rate of 94.74%. MRSA had the highest reinfection rate at 20.75% (Figure 2). Those with greater than 5-year follow-up, 6/11 reinfections were due to the same organism (MRSA-2, culture negative-2, Psuedomonas-1, MSSE-1, Enterococcus-1, and E.coli-1). Of those with 2–5-year follow-up, 4/5 were reinfected by same organism (MSSA-2, MRSA-2). Those with <2 years follow-up, 8/13 were reinfected by same organism (Culture negative-5, MRSA-2, Coagulase negative staph-1).

### 3.1. Short-Term (0–2 Years) Follow-Up

Results showed that 58 patients were identified with less than 2-year follow-up. This included failure of reimplantation and mortality before two-years following the first-stage procedure. Average follow-up was 9.56 (range, 0.10–24.00) months. Successful eradication was determined to be 94.12% with mortality rate of 9.95% (22/221) (Table 2). 

### 3.2. Mid-Term (2–5 Years) Follow-Up

Results showed that 34 patients were identified with 2–5-year follow-up. This included failure of reimplantation and mortality between 2–5 years following the first-stage procedure. Average follow-up was 3.72 (range, 2–5) years. Successful eradication was determined to be 98.77% with mortality rate of 9.20% (15/163) (Table 2). 

### 3.3. Long-Term (5+ Years) Follow-Up

Results showed that 129 patients were identified with greater than five-year follow-up. This included failure of reimplantation and mortality before two-years after first-stage procedure. Average follow-up was 9.19 (range, 5–25.5) years. Successful eradication was determined to be 91.47% with mortality rate of 41.10% (53/129) (Table 2). Three patients did not receive a reimplantation. Two patients were revised due to instability and periprosthetic fracture. 

### 3.4. Survival

**Figure 3:**
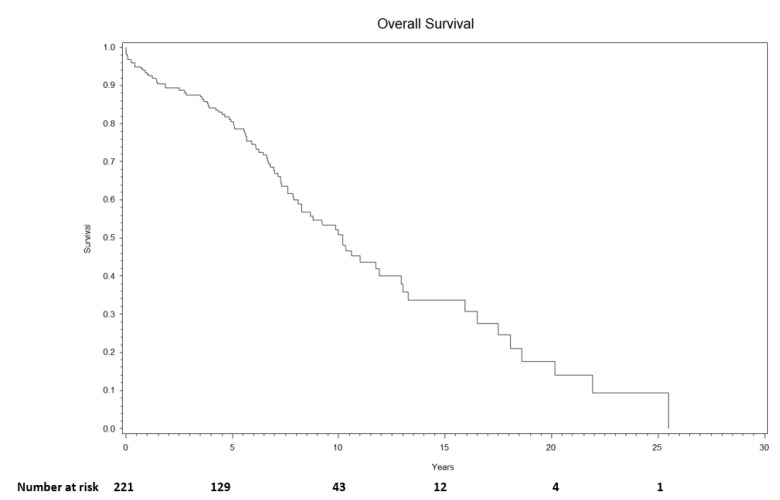
Overall survival following two-stage exchange at 1-year, 3-year, and 5-year was 93% (95% CI: 89–96%), 88% (95% CI: 82–91%), and 80% (95% CI: 74–86%), respectively.

**Figure 4:**
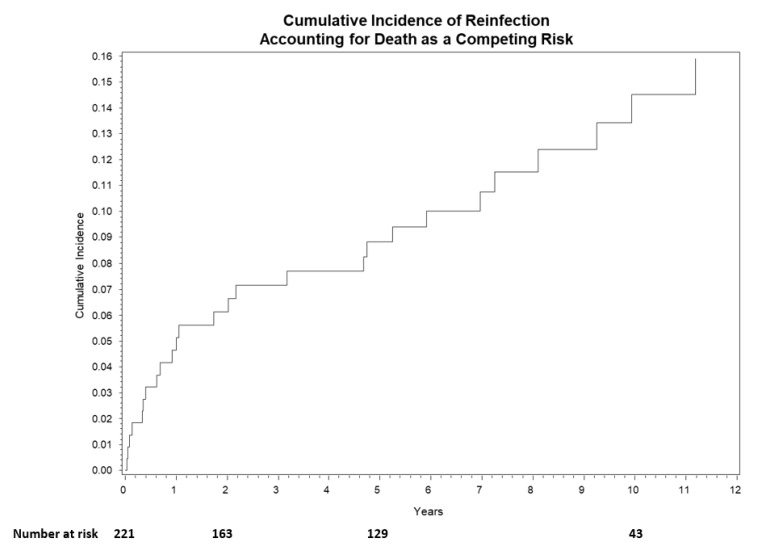
Cumulative incidence curve for reinfection. Overall incidence at 1-year, 3-year, and 5-year was 5% (95% CI: 2–8%), 7% (95% CI: 4–11%), and 9% (95% CI: 5–13%), respectively.

The median follow-up of surviving patients was 5.80 (range, 0.40–23.40) years. Overall survival at 1-year, 3-year, and 5-year was 93% (95% CI: 89–96%), 88% (95% CI: 82–91%), and 80% (95% CI: 74–86%), respectively (Figure 3). Cumulative incidence of reinfection at 1-year, 3-year, and 5-year was 5% (95% CI: 2–8%), 7% (95% CI: 4–11%), and 9% (95% CI: 5–13%), respectively (Figure 4).

### 3.5. Risk Factors

Univariate analysis identified two covariates associated with increased risk of infection. Risk for reinfection for patients with a polymicrobial infection identified by culture was 2.63 times higher than patients with one organism (95% CI: 1.17–5.91, *p* = 0.019). Antibiotic resistant organism was associated with a 2.36 times higher risk of reinfection (95% CI: 1.1–5.04, *p* = 0.027). In a multivariate analysis, the risk of reinfection was significant for patients with polymicrobial infection (HR = 2.36, 95% CI: 1.08–5.14, *p* = 0.031). Risk factors not identified for reinfection included: diabetes, smoking, systemic disease, chronic kidney disease, and previous number of two-stage procedures (Table 3). Average number of major comorbidities per patient was 2.55 (range 0–11). There were 174 (78.70%) patients with at least one major comorbidity. Of those who were reinfected after two-stage exchange, patients with one or more comorbidity had a failure rate of 80.80% (21/26). 

## 4. Discussion

This is the largest study evaluating outcomes in subjects with more than five-year follow-up after undergoing a planned two-stage exchange for chronic PJI. We report an infection eradication rate of 88.24% with an average 6.14-years follow-up. The infection eradication rate was 91.47% in subjects with more than five-year follow-up. The mortality rate was 41.1%. The most common organism was coagulase-negative staphylococcus.

Mortality remains relatively high despite overall excellent infection eradication rates. In 2018, life expectancy for males and females was 76.2 and 81.2 years, respectively, with overall death rates between ages 65–74 at 1783.3/100,000 population (1.78%) [16]. These rates are similar among patients with primary TJA and matched cohorts with osteoarthritis in the first 10 years, but marginally rise thereafter [17,18,19]. To compare, overall cancer, uterine, breast, and prostate have a 5-year survival rate of 67.4%, 81.2%, 90.0%, and 97.8%, respectively. This compares to 80% in patients who underwent two-stage exchange for chronic PJI [20]. Higher probability of poorer hosts, morbidity of the treatment and nature of infection, and medical complications in this patient cohort may also play large role in the reported mortality rates. Not only should we counsel patients on success rates, but also risk of death following this treatment pathway. 

There is paucity of data reporting long-term outcomes following two-stage exchange arthroplasty. Petis et al. described a reinfection rate of 15% and mortality rate of 56% in 164 hips with average follow-up of 12 years at a single institution [13]. However, there were 49% of the original cohort of patients that were excluded as they received prior treatment for PJI. This likely introduced selection bias. We attempt to limit selection bias by not excluding patients with prior treatment for PJI and combining results of three large tertiary referral centers. A study out of the Danish Registry reported a 5-year reinfection rate after reimplantation of 14.6% (95% CI: 8.0–23.1) with overall survival rate of 68% (95% CI: 59–75) [21]. In a systematic review and metanalysis by Lange et al., they estimate risk of reinfection following two-stage exchange at 10.4% (95% CI: 8.5–12.7%) [22]. A single-surgeon retrospective review of 155 hips resulted in overall 91.7% survival rate with an average of 9.7 years follow-up and 16.1% mortality rate [14]. Overall, our results are similar, however we reported a much higher mortality rate. This may be attributed to overall health of patients treated at the three large tertiary referral centers and geographic differences in overall health of patients in each study. Knutsor et al. revealed an unadjusted reinfection rate of 32.3% amongst pooled data of 1856 two-stage hips from 44 cohorts with average follow-up of 3.7 years. Infection rates for studies including over 50 hips is between 1.7–10.7% [23,24,25]. Triantafylloppoulos et al. reported 91.24% infected eradication rate in 548 patients treated with two-stage exchange with minimum two-year follow-up [26]. They identified heart disease, psychiatric disease, and female gender as a risk factor for failure. Despite previously reported comorbidity risk factors for failure, based on our multivariate regression analysis we did not identify any significant risk factors [9,10]. However, our results are similar to more recent literature suggesting polymicrobial and/or resistant organisms predict higher failure rates [12]. Host grade was not documented consistently at our institutions although we support previous reports indicating host grade as a significant risk factor [11]. Most of our cohort (78.7%) had one or more major medical comorbidity with average of 2.55. Because many patients who unfortunately sustain PJI’s have underlying major medical comorbidities, identifying one major comorbidity over another as a risk factor is often difficult. 

We report a reinfection rate of 8.53% in patients with more than five-year follow-up. The ongoing risk of reinfection after five years should be further explored and re-iterated to patients. Despite the relative urgency to treat infection, patient optimization would likely decrease mortality risk and improve infection eradication. Future research investigating the routine use long-term antibiotic suppression is essential. 

Patients with MRSA had a failure rate at 20.75%. This resistant organism has been previously reported as an independent risk factor with reinfection rates of 21–38% [6,7]. Suboptimal antibiotics and inability to maintain minimal eradication concentrations make these resistant organisms difficult to treat. Failure rate of polymicrobial infections was also high at 21.9% (including infections with MRSA) as has been reported [27,28]. This can be explained by difficulty maintaining treatment with broad spectrum antibiotics and increased risk of resistance. Although there is no data to our knowledge evaluating reinfection patterns, short-term failure may be resultant of more virulent bacteria while later failure by more indolent organisms. We did not identify this pattern as there was a mixed of more indolent and virulent bacteria despite the longevity of follow-up. We report a relatively high success rate when treating culture negative infections (93.55%) similar to that reported by Haddad et al. (94%) with greater than five-year follow-up [29]. We did report a high proportion of patients with culture negative infections. This may be explained by not excluding patients with prior PJI treatment or who were maintained on antibiotics prior to treatment. Without antibiotic sensitivity, these infections are difficult to treat; however, outcomes appear favorable with standardized two-stage exchange protocol and broad-spectrum antibiotics. Future endeavors to improve diagnostics and antibiotic sensitivity identification may improve success rates. 

Aside from infection, we report a total complication rate following patients who underwent a first stage in a planned two-stage of 14.93% (33/221). Adjusting for subjects who did not proceed to reimplantation, we report a dislocation rate of 2.45% which is significantly lower than the one-year cumulative risk of dislocation of 9% (52/512) identified by McAlister et al. [30]. This study however did not use reoperation for dislocation as endpoint and included patients who also failed due to infection simultaneously. Other studies consisting of a minimum of 50 hips report similar dislocation rates (3.4–4.4%) and fracture rates (1.5–3.4%) [23,24,25]. 

This study has several limitations. There was no standardized method to determine infection eradication prior to reimplantation. The study retrospectively spanned 25 years where perioperative management, diagnosis, and treatment techniques have evolved. The use of MSIS criteria was generally used to diagnose infection after 2011. Prior to this date, diagnosis was determined clinically by the treating surgeon. Despite the variability in this study cohort, the overall outcomes were similar between institutions. We also attempted to limit selection bias with substantial follow-up until reinfection or death. Unfortunately, documentation of variables that have been reported as risk factors for reinfection such as extremity grade, duration and concentration of antibiotics, and interim time between stages were not recorded [11,31]. The retrospective landscape of this report across three institutions inherently contains bias and may decrease the quality that is dependent on data reporting and mining. Functional outcomes were not reported. Reoperation was used to define failure. This likely overestimates success rates specifically if a complication, persistent pain, and/or debilitation did not have a surgical remedy. The results are likely best-case scenarios. 

Periprosthetic hip infection remains as a morbid complication with a relatively high mortality rate despite the reported success rate. Polymicrobial and resistant organism infections are difficult to treat with high reinfection rates. We reiterate the importance to educate surgeons and patients on the persistent long-term risk of reinfection in addition to the relatively high mortality rate. Improving patient optimization, decreasing surgical morbidity, and enhancing therapeutic and diagnostic regimens should be further explored in this patient cohort. 

## Figures and Tables

**Table 1:** Outcomes. Non-infection failure refers to mechanical failure requiring operative intervention.

Outcomes-Cumulative	N	%
All-Cause Complication 1-and 2-stage	43/221	19.45
Failure Rate-Infection after 2-stage	26/221	11.76
Failure Rate-Total after 2-stage	41/221	18.55
Failure Rate-Non-Infection after 2-stage	7/221	3.17
Failure Rate-Infection after 1-stage *	14/221	6.33
Failure Rate-Total after 1-stage **	18/221	8.14
Failure Rate-Non-Infection after 2-stage	4/221	1.81
Failure to Reimplant	30/221	13.57
Failure to Reimplant after 1 year	17/221	7.69
Total Mortality	90/221	40.72

* Eight went on to success. ** 10 went on to success.

**Table 2:** Outcomes by follow-up.

Outcomes by Follow-Up	0–2 Years	2–5 Years	5+ Years (5–25.5 Years)
N	58/221	34/163	129
Average Follow up (Months)	9.56	44.68	110.23
Mortality	9.95% (22/221)	9.2% (15/163)	41.1% (53/129)
All-Cause Failure (2 stage)	8.14% (18/221)	6.13% (10/163)	10.08 (13/129)
Failure 2-stage infection	5.88% (13/221)	1.23% (2/163)	8.53% (11/129)
Remaining 1-stage	8.14% (18/221)	5.52% (9/163)	2.3% (3/129)
Average Age	64.68	67.87	65.73
Total average follow-up (months)			73.72
Total mortality			40.72%

**Table 3:** Associated and non-associated variables. Abbreviations. MI: myocardial infarction, CHF: congestive heart failure, and DVT: deep vein thrombosis.

Variable	HR	95% CI	*p*-value
Polymicrobial	2.63	1.17–5.91	0.0194
Resistant Organism	2.36	1.10–5.04	0.0273
Sex	0.539	0.244–1.19	0.1264
Previous 2-stage	1.37	0.327–5.731	0.6673
Diabetes	1.56	0.673–3.62	0.299
Chronic Kidney Disease	1.517	0.58–3.97	0.3952
Coronary vascular disease	0.42	0.099–1.78	0.2395
Prior MI	0.731	0.096–5.576	0.7628
CHF	0.684	0.095–4.904	0.7054
Prior DVT	1.466	0.574–3.745	0.4239
Current smoker	1.029	0.393–2.694	0.9529
Former smoker	0.445	0.134–1.486	0.1882
Systemic Disease	1.332	0.489–3.631	0.5755
Immunocompromised	1.785	0.450–7.085	0.4099

## Data Availability

No new data were created or analyzed in this study. Data sharing is not applicable to this article.
